# Navigating power imbalances in research with children and young people in shelters for domestic violence

**DOI:** 10.1177/14733250261444083

**Published:** 2026-07-02

**Authors:** Michèle Frenette, Simon Lapierre

**Affiliations:** 1 School of Social Work, 6363 University of Ottawa, Ottawa, ON, Canada

**Keywords:** domestic violence, children, ethnography, power, feminism, shelters

## Abstract

This paper examines the methodological approach from a doctoral research project based on an ethnography conducted with children and young people living in shelters for domestic violence. Guided by the principles of critical social work research, this study was co-constructed in partnership with a feminist umbrella organization representing shelters across the province of Quebec, Canada. This collaboration ensured that the research had meaningful outcomes for both theory and practice. In this paper, I discuss how methodological and theoretical decisions worked to reduce power imbalances throughout the research process, emphasizing the critical role of interpersonal relationships in data collection. Drawing on feminist care theory (Brannelly and Barnes, 2022) and principles of feminist intervention in shelters (Côté, 2017), I highlight the importance of play, adopting a “least adult” posture (Mandell, 2003), supporting the mothers and a child-centered approach to establishing connections and navigating traditional power dynamics. This approach enabled a deeper understanding of the children’s and young people's lived experiences in shelters. The findings suggest that research with children and young people necessitates adapting traditional research methods to better align with their realities. This requires critical reflection and proactive actions - such as thoughtful methodological and theoretical choices - to address power imbalances between adult researchers and child participants.

Research about children living in the context of domestic violence has evolved significantly over the past 50 years Lapierre et al., 2025. In her literature review, Øverlien (2010) noted that most studies on children experiencing domestic violence were conducted in the field of psychology, used quantitative methods, and relied on mothers to gather information about the children’s experiences. She recommended conducting qualitative studies that prioritize children’s own perspectives.

More recently, Lapierre et al., 2025 conducted a systematic review of children’s participation in these contexts, highlighting a shift toward qualitative research that gives greater importance to children’s voices, as well as a diversification of research fields. Thunberg et al. (2024) also emphasize the evolution of research on domestic violence, using a child-centered approach, and note improvements in the conditions of children in shelters. Thunberg et al. (2024) observe that while shelters primarily prioritize children’s right to protection from violence, they also highlight the need to support children’s rights to assistance, education, leisure activities, and social connections to enhance their living conditions in shelters. The authors further stress the need to further document children’s experiences and perspectives, both in research and in social work practice.

In this same perspective, Côté et al. (2020) discuss a more general shift in paradigm and epistemology in research involving children, moving away from a positivist approach to instead adapting methodologies to children rather than the other way around. However, conducting research with children presents unique challenges. For example, Corsaro (2015) points out that accessing children’s worlds is challenging due to the physical size of adults and the power they hold over children, which reinforces the perception of adults as authority figures. Similarly, Côté et al. (2020) emphasize the importance of researchers engaging in a reflexive and critical process regarding their role, preconceived notions, and methodological choices, particularly to reduce power imbalances between adults and children.

This paper aligns with this significant evolution in research on children living in the context of domestic violence. It is based on a doctoral study in social work focusing on the experiences and perspectives of children and young people^1^ in shelters for women victim of violence and their children. More specifically, the goal of this paper is to demonstrate how my^2^ methodological approach, based on ethnographic research with children and young people in shelters, helped reduce power imbalances between myself and the children and young people.

This paper is divided into six sections. First, I will present a review of the literature on the experiences of children living in shelters. Second, I will introduce the theoretical framework of the new sociology of childhood, including my epistemological stance rooted in critical and emancipatory social work theories. Third, I will describe my methodological approach, starting with a practicum. The decision to focus on children in shelters arose from this practicum and shaped my methodological choices, particularly ethnography, which will be covered in this section. Then, I will share excerpts from my field journal to illustrate my investment in the relationship with children and young people within my research setting that contributed to reducing power dynamics. Finally, I will discuss the methodological choices I adopted, supporting them with feminist care theory and the principles of feminist intervention in shelters, and I will conclude by addressing the implications of these choices for research and practice with children in shelters.

## Research on children and young people living in domestic violence shelters

Research on children’s and young people's experiences in domestic violence shelters has primarily relied on individual and group interviews, or interviews with mothers and shelter staff (Chanmugam, 2011; Mullender et al., 1998; Thibault, 2021; Øverlien, 2011). Several recurrent themes emerge across these studies. Play and the availability of play spaces were identified as essential for children’s positive experiences in shelters (Mullender et al., 1998; Thibault, 2021; Øverlien, 2011). Feeling heard and safe was highlighted as a central positive aspect of shelter life (Chanmugam, 2011; Mullender et al., 1998; Thibault, 2021; Øverlien, 2011). Peer relationships were another important dimension: young people appreciated building friendships with other residents (Chanmugam, 2011; Thibault, 2021), and collectivizing their experiences contributed to a sense of empowerment (Thibault, 2021). However, transitioning into shelters presented challenges, particularly regarding shelter rules and admission (Chanmugam, 2011; Sopczyk, 2007; Thibault, 2021). Some children, including young people, children with disabilities, and Black children, were noted as receiving insufficiently adapted services (Chanmugam, 2011; Mullender et al., 1998). Overall, these studies conceptualize shelters as not only spaces of safety but also as intervention sites that should actively foster empowerment, play, and well-being (Øverlien, 2011; Sopczyk, 2007).

While existing research has made important contribution to better understand children’s and young people's shelter experiences, these studies rely primarily on interviews with children and the adults around them. Although these studies emphasize the perspectives of children and young people, their methodological frameworks are constructed around adult perspectives and prioritize a single form of participation such as interviews thereby limiting access to children’s everyday practices, their interactions with other children and adults and their roles as social agents.

Sopczyk (2007) ethnographic study provides valuable insights within this body of literature. Rooted in a postpositivist approach, Sopczyk (2007) conducted participant observation and formal and informal interviews with women, children and staff in shelters. In her research, children are mostly seen through frameworks of vulnerability, adaptation and developmental outcomes. As a result, children’s agency remains invisible.

## Theoretical framework and epistemological positioning

Building on Sopczyk’s methodological contribution to ethnographic work in shelters, this study draws on the new sociology of childhood to critically examine children’s agency and power relations within shelters. My critical theoretical stance further offers a reflexive perspective on the researcher’s role with children, contributing to a deeper, more engaged understanding of their experiences and addresses ethical components of doing research with children.

### The new sociology of childhood

In social sciences research, the recognition of the importance of including children’s voices emerged in the late 1970s, particularly with the development of childhood studies (James & Prout, 2015). The adoption of the Convention on the Rights of the Child in 1989 further strengthened their right to participation, promoting research conducted *with* children rather than *on* them, thus moving away from more positivist methodologies (Lavoie et al., 2020). This epistemological shift requires adults to adapt to children’s realities rather than the other way around, redefining research practices to center on children’s voices and experiences.

The theoretical framework of the New Sociology of Childhood (hereafter NSC) (Prout & James, 2015) aligns with my research project due to the following four principles: 1)The child is recognized as a social actor; (2) Children’s agency and ability to influence their environment are acknowledged; (3) Power dynamics in research are identified and critically examined; (4) Participatory methodologies like ethnography are essential to promote children’s participation and access their perspectives.

#### The child as a social actor

It was during the 1980s-1990s that the NSC was developed (James et al., 1997). The pioneers of this theory observed that children were invisible in sociological research (James et al., 1997; Qvortrup, 2005) and was developed through a constructivist epistemology (Prout & James, 2015). This constructivist perspective suggests that children are competent social actors and that their viewpoints are valid, even necessary, in understanding their reality in the present moment (Hamelin-Brabant, 2006).

#### Recognition of children’s agency

Another key concept of the NSC is agency, which views children as competent social actors capable of making sense of and acting upon the world (James, 2001). In research, this translates into seeing children as competent social actors independent of adults (James & James, 2001). Children are not passive; rather, they are active in constructing their world and in how they make sense of their experiences as children (James & James, 2001). According to this view, the child is not only capable of expressing opinions and desires about their world but also has the right to do so, as outlined by the Convention on the Rights of the Child.

#### Power relations

James & James (2001) discuss the control adults have over children’s behavior and, consequently, over their childhood. If the child is seen as vulnerable - according to most positivist and developmental theories - the adult is thus perceived as having a protective and controlling role over the child (Mayall, 2000). This control maintains a certain status quo regarding what constitutes childhood and the place of children in society (James & James, 2001). However, if the child is seen as a citizen with rights, it encourages a more egalitarian role between the child and the adult (Mayall, 2000).

#### Ethnography: Making children’s experiences visible

One of the principles advanced by the pioneers Prout & James (2015) is that ethnography is a method to be prioritized in research with children, as it allows access to the children’s world through their daily actions from their own perspective. James & James (2001) argues that it is through ethnography that researchers have contributed to the paradigm shift regarding children in research, moving them from invisible to visible. James (2001, p. 247) states that:

“Ethnography expressly facilitates the desire to engage with children’s own views and enables their views and ideas to be rendered accessible to adults as well as to other children”. Corsaro (2015) supports that ethnography is an appropriate methodology for children, as observing daily life provides direct access to the children’s world in the present moment.

### Epistemological stance

This research project is grounded in critical and emancipatory theories in social work, which analyze how power relations influence society and create injustices and oppression for certain groups. These theories oppose the positivist paradigm, which advocates maintaining a distance from the research “subject” to avoid the researcher influencing the results (Potts & Brown, 2015). Objectivity is essential in this paradigm, as the researcher must not be influenced by personal opinions, identities and biases. (Potts & Brown, 2015). However, feminist standpoint theorists, such as Haraway (1988) and Harding (2004), have questioned the concept of objectivity in research. According to them, research is influenced by gender, race, social class, and other aspects of the researcher’s identity, in this case, age. The production of knowledge often reflects the perspectives of privileged groups (Haraway, 1988; Harding, 2004), in this case, adults.

Thus, this research requires a reflexive posture with my own standpoint as an adult doing research with children. As an adult researcher, and parent, trained in social work specifically on domestic violence, I entered the field with assumptions influenced by my standpoint and identities regarding children’s participation, adult-child relationships, children’s behavior and shelter life. To challenge these assumptions and make space for children’s lived experiences, questioning adult judgements about parenting practices, resisting tendencies to question or comment on children’s play or screen activities and being attentive to children’s non-verbal actions such as play, relationships, silences and their presence within the shelter.

This reflexive posture also required attending to children’s standpoint as a source of knowledge. As an oppressed group in a space where adults hold most of the power, children have epistemic privilege regarding their everyday experiences of shelter life. Focusing on children’s perspectives through play, non-verbal communication, and everyday practices allowed access to knowledge about their experiences in shelters and within intervention contexts.

Influenced by critical and emancipatory theories who critique power relations in knowledge production, deconstructing my adult standpoint was primarily done by prioritizing proximity with participants. This involved how I showed up with children by letting them set the pace of our relationship, following their lead in terms of activities and discussions and refraining from commenting on their behaviors, activities choices and how they expressed themselves. Critical approaches challenge hegemonic norms in research and advocate for participatory methods that start from the perspective of those directly involved in the research. This ensures that the knowledge produced genuinely reflects their experiences (Haraway, 1988).

## Methodological approach

### Practicum

Since my doctoral research is grounded in critical social work and I wanted the research to have impacts both for research and practice, I chose to undertake a practicum^3^ as part of my doctoral journey. Interested in feminist intervention in shelters, I approached a provincial feminist umbrella organization to co-construct a research project. This non-profit organization brings together and supports a network of shelters for women who have been victims of violence and their children. The organization plays an advocacy and awareness role regarding violence against women while supporting its member shelters.

The practicum allowed me to consult with this organization and its members to learn more about one of their most recent projects, which aimed to integrate intersectional feminist intervention within their member shelters. They observed that women who entered shelters faced various challenges in addition to experiencing domestic violence (e.g., substance abuse or mental health issues). They also realized that interventions for women dealing with multiple issues could create challenges, particularly in building alliances with them. The organization decided to engage in a reflective, political, and theoretical process to work towards more inclusive practices using an intersectional feminist approach.

The consultations and my involvement in various activities within this organization during my practicum demonstrated that efforts to integrate intersectional feminist intervention into shelters had not yet been translated into interventions for children. In line with my theoretical framework, this study aimed to understand the experiences of children and their perspectives on these practices. Drawing from qualitative methodologies and aligned with the NSC, ethnography was chosen as the method for data collection.

### Ethnography

Ethnography, as it draws on multiple data collection methods such as observation, interviews, and participation in daily activities (Woodward, 2019), allows for a deeper understanding of a phenomenon. Moreover, due to its inductive approach, ethnography offers flexibility to adapt to the realities on the research field and, by extension, to the primary subjects of the research - the children (Corsaro, 2015). Ethnography is a methodology that prioritizes proximity to participants, particularly through participant observation (Woodward, 2019). As Scott (2009, p. 193) states, “the most obvious way of understanding somebody else’s everyday life is to go to their locale and experience it directly.” Therefore, ethnography is an immersive experience that prioritizes relationships with people involved in the research settings (Woodward, 2019). This proximity was crucial for reducing power imbalances and gaining access to the experiences of children in shelters.

Access to the research field (shelters) was achieved in collaboration with the feminist organization. Following my practicum, a presentation was made to all the member shelters to introduce the proposed research project, and it generated significant interest. The next step was to approach a first shelter, chosen for its geographical proximity, making weekly travel for participant observation with the children easier. A meeting was held with the shelter’s director, coordinator, and youth worker to explain the project and the implications for the shelter and the children residing there. Due to the frequent turnover of families in shelters and the uncertainty about the length of their stays, a second shelter was approached. As with the first shelter, I met with the director and presented the research project to the team, who agreed to be part of the research project.

The mission of the first shelter, located in a more urban area, is to accommodate a maximum of 9 people and support women who are victims of domestic violence and their children. Situated in a residential neighborhood, this shelter has two floors, with rooms on both the first and second floors. The offices for the shelter workers are also spread across both floors. There is a reception desk at the entrance, and the living room and kitchen, where most of the women and their children spend their time, are also located on the first floor. However, children are not allowed in the living room on the first floor because it is dedicated to the women in the shelter. They do, however, have a playroom downstairs. There is also an outdoor courtyard with play equipment. I spent most of my time in the kitchen, the playroom, and the outdoor courtyard.

The second shelter is in a suburban area, with a capacity of 10 beds, and it accommodates women who are victims of violence or facing temporary difficulties, along with their children. Unlike the first shelter, this second one can also house women dealing with various issues (mental health, substance use, homelessness, violence). This choice allowed for a more enriching and diverse experience in terms of feminist intervention. This shelter was in a slightly more isolated and remote area. It had three floors: the ground floor had bedrooms, a kitchen, and a living room in the same space, where most of the women and their children spent their time, as well as the reception desk. On the lower level, there were offices for the shelter workers and a playroom for the children, which was rarely used due to its location in the house. The upper level contained additional offices and meeting rooms. There was also an outdoor courtyard with play equipment for the children. I spent most of my time in the kitchen and the living room, where the children were most often.

In the first shelter, I initially spent time at the shelter to familiarize myself with the environment and to talk with the shelter workers, especially the youth worker who would become an important ally in connecting with the children and their mothers. Since a key component of ethnography is being present and conversing with participants (Woodward, 2019), I started visiting the shelter at different times during the week (days, evenings, and weekends) to assess the best moments to be with the children. It was during a cooking activity organized by the shelter workers, in which I participated, that facilitated my integration. I was able to informally talk with the children and their mothers, introduce the research and explain my role in a fun and relaxed environment. I participated in organized activities, and I spent a lot of informal time with the children: playing in the playroom, playing outside, chatting, and doing activities in the kitchen.

For the second shelter, I chose to be present when the youth worker was working in the evening, and the children were also present. This allowed me not only to gain access to the children through her, but also to observe the interventions being made with the children, her role. I participated in activities organized for the children outside the shelter, such as a special event for International Children’s Rights Day, and I spent time in the shelter with the children: playing in the kitchen, in the living room, talking to them, and participating in activities organized for special celebrations.

While shelter workers, particularly youth workers, often acted as allies by introducing me to the children and including me in activities, their presence also shaped my access in different ways. When workers were more present, opportunities for interaction with children were sometimes more limited, although these moments allowed for observation of interventions and workers roles. In contrast, when I was alone with the children in shared spaces, opportunities for informal exchanges and relationship-building were more frequent, facilitating deeper connections with the children and young people.

As Woodward (2019, p. 128) notes: “The relationship between observing, participating, and talking shifts and often emerges organically.” This describes my experience at the shelter: between participating in activities and daily life at the shelter (I did cleaning, had meals with the families, supported the mothers), observing, playing, talking with the children, mothers and shelter workers, and taking notes. In total, I conducted participant observation with children aged 0 to 17 in both shelters for six months.

### Data collection and data analysis

#### Writing down

O’reilly (2012) discusses “writing down” and “writing up” in ethnography. “Writing down” refers to taking notes and collecting information through observation and transcribing informal conversations. Much of the work involves describing observations and exchanges. I documented the activities, conversations, what I was doing, the games, what the shelter workers were doing with the children, and my thoughts and emotions. I also took photos of various activities we did and kept things that the children gave me, such as drawings.

When observing children, researchers need to be cautious to minimize adult biases and highlight the daily lives and experiences of the children (Corsaro, 2015). Although I tried as much as possible to directly describe the children’s experiences and gather their perspectives, while also relying on the new sociology of childhood as a theoretical and empirical lens in data collection, I cannot claim to have described and analyzed the data without any bias. However, the methodological and theoretical choices discussed in this text have hopefully facilitated access to the children’s world.

#### Data analysis

O’reilly (2012) further explains that when we feel we have collected enough information through notetaking and data gathering (“writing down”), it is time to analyze the information by organizing the notes and “making sense of it all” (p. 186). She notes, however, that in ethnography, these steps are interconnected, a process she calls an “iterative-inductive approach” (p. 180). “This is a practice of doing research, informed by a sophisticated inductivism, in which data collection, analysis, and writing up are not discrete phases, but inextricably linked.” (p.180). O’reilly (2012) emphasizes that analysis involves reflection throughout data collection, with back-and-forth between reflections, data, and theory. I indeed reflected continuously throughout my data collection, so much so that I coded my field journal by separating observations and reflections. Here are some examples of reflections I noted in my journal:

When saying goodbye to worker 2, I tell her it was a nice evening and thank her. She says she doesn't know what activity to plan for November – I feel like saying: why not ask the children? But I hold back. Couldn't there be an opportunity to ask the women and children at the shelter if there are any celebrations from their cultures and organize an activity around that? Reflection on the need to return to basics, simplicity with the children: just ask them.

This moment highlights a tension in two ways. First, my desire to suggest that the worker ask the children what they wanted to do reveals a tension between my theoretical framework, which emphasizes children’s participation and agency, and my awareness of my role as a researcher within an intervention context. I chose not to intervene to avoid overstepping the worker’s role, being aware of the potential consequences for our relationship. This hesitation also highlights how children’s participation can be easily overlooked, and how their exclusion from everyday decisions can shape their experiences in the shelter.

These reflections show that the construction of analysis took place throughout data collection and was directly influenced by what happened at the shelter with the children. I was already reflecting on children’s experience at the shelter, interventions and the role of the adult in the children’s daily lives in the shelter.

To make sense of the data, I first reread my notes. Then, it was necessary to move the data out of their chronological order and organize them by themes (O’reilly, 2012). I began by coding in Nvivo into two broad categories: the children’s experience at the shelter and the ethnographic experience. The final step is “writing up,” which means organizing the field journal notes in a way that presents them.

## Results

It was during the analysis stage that I realized I had taken many notes related to the ethnographic experience within the shelter. It was also at this stage that I recognized how much time I had invested in building relationships with the children, their mothers and the shelter workers. I also became aware of how often I had documented my various emotions related to the ethnographic experience. For example, when the families left to live in their new housing: *“*I said goodbye to Mother 2 and Child 3, who are moving this week. It was bittersweet. I felt a little sad; I had gotten attached to them. Playing together on Mondays, chatting with them.” This quote illustrates the connections that are made in a living environment context, like ethnography where time is spent together over a given period, sharing in their intimacy. This section is therefore dedicated to describing the investment in relationships with the children through excerpts from the field journal.

### Fostering connections with children through play, interests, and everyday moments

In this section, I explore the different strategies I used to create relationships with children living in shelters. The sub-sections present four ways connections were formed: through play, by engaging with young people’s screen activities, by supporting younger children through their mothers, and by recognizing and valuing signs of connection initiated by the children and young people.

#### Play

To build connections with the children and gain insight into their daily lives in the shelter, I spent a lot of time playing with them. The following quote illustrates what the evenings looked like in this context. The younger children would often ask me to play with them in the designated playroom. They particularly enjoyed building “obstacle courses” and playing music. I was there with them, playing, singing, and dancing. This way of doing research seemed to contradict more traditional approaches, as one of the mothers pointed out when she saw me playing with the children:

We played for a long time in the playroom: jumping, making obstacle courses. Child 3 asked me to play ‘the floor is lava.’ We sang, we danced. Mom 1 looked at me and said, laughing: ‘Does that help you with your research?’ I responded: ‘Of course!’

Play was not only a great way to build relationships with the children, but also an important methodological tool. Through play, I had access to ways children express themselves that doesn’t rely on verbal communication like in interviews. Following Mandell (2003) “least adult” role*,* engaging in play created a space where children could lead the interactions and build a relationship with me outside the adult-authority-role. When the mother asked if this was helping my research and I responded “of course,”, this was a way to normalize my presence in the children’s everyday activities, mostly through play. This was also an opportunity to indicate to the mother and the children that I was willing to participate in their ordinary interactions rather than maintain a distant researcher role. This was also a great way to observe how children interact with each other and how they take space in the shelter which made visible aspects of their everyday lives.

In the second shelter, there was a group of three brothers: one younger and two teenagers with whom it was more difficult to establish a connection. The two young people were often occupied with extracurricular activities or stayed in their room, so they were rarely present in the common areas of the shelter, making interactions with them more challenging. One evening, I suggested playing cards with the youngest (one shelter worker had told me that he liked to play the card game Uno), and he agreed. His two brothers eventually joined us. This game moment became a great opportunity to build a connection not only through play but also by discussing other subjects, all while maintaining a playful and relaxed atmosphere.

Adopting a “least adult” approach (Mandell, 2003) was particularly useful and effective in this situation. It allowed me to not exercise adult authority over the children, such as criticizing their language, certain comments they made or the music they were listening to. My role was simply to be present with them, to integrate into their world without judgment.

This connection also became a source of happiness for me, as described in my journal notes:

I asked Child 4 if he wanted to play cards, and he said yes!!! Victory! Then Child 5 joined us... We played for an hour; it was enjoyable. I felt like we connected, we laughed a lot, made jokes, the atmosphere was very relaxed and fun... I took the opportunity to chat a bit more and talk to them about school... Child 6 joined us for the last game. Child 4 took his headphones and put them on my ears so I could listen to Child 5’s music. Really a great evening to connect... super pleasant. I’m happy with my evening.

This moment highlights how adopting a “least adult” posture created space for informal and spontaneous exchanges initiated by the young people. They talked about school, music, and everyday interests outside of the expectations often associated with research objectives, typically via interviews. This allowed me to access young people’s perspectives in the present moment through ordinary conversation, during a typical evening of playing cards in the shelter.

#### Connecting through screens: Engaging with young people on their terms

In addition to play, I also sought to engage with the children and young people by showing interest in what they were doing on their screens. Again, adopting the “least adult” approach (Mandell, 2003) was valuable as it allowed me not to comment on their screen usage or suggest other activities but rather to genuinely engage with their activities, with the goal of creating a connection. While this approach was useful with children of all ages, I wish to emphasize its particular importance with young people: “I chat with Child 6 about her TikTok videos. I take the opportunity to chat a little with her about her stay so far.” Starting the conversation about her social media and being genuinely interested in what she was listening, allowed me to get to know her a bit more and gave me an opportunity to learn more about her world. This also created a familiar atmosphere which she could then talk to me about how she found it difficult since arriving in the shelter because the playroom was mostly for younger children and she was not allowed to access the living room since it was reserved for the women. This aligns with existing literature about adolescents in shelters and how it is difficult for them to have their own space (Chanmugam, 2011; Mullender et al., 1998).

#### Connecting with younger children by supporting their mothers

Connection with younger children was primarily made through supporting their mothers. Research shows that support for the mother plays a key role in the safety and well-being of the children (Lapierre, 2019; Mullender et al., 1998; Thibault, 2021). By offering a space for listening and collaboration with mothers, I was able to gain access to younger children’s everyday lives in shelters and to connect with them:

Child 11’s mother asks me to watch her while she goes to get something. I play with her on the floor, she laughs.

Mother 3 tells me she tried to shower three times today, but she [ the baby] always wants to be held. I offer to hold her while she showers. She’s so happy. She tells me she’ll enjoy it while I’m here.

The support provided to the mothers and the time spent chatting with them also constituted a central aspect of my fieldwork. Because younger children are more often with their mothers in shelters, building trust with mothers allowed me to access their children. This was both an ethical and methodological component of the research that allowed access to younger children’s everyday experiences in the shelter.

### Recognizing connection

As mentioned earlier, play turned out to be an essential method for establishing contact and building connections with the children. Additionally, certain gestures from the children showed they were increasingly recognizing my presence and appreciating our interactions. Connection was not always immediate. At times, children chose not to engage or even asked me to leave while playing together. Respecting these boundaries and letting children initiate interactions on their terms led to, eventually, moving from disconnection to connection. This manifested, for example, in the nicknames they used for me and their initiative to suggest games to play with me:

We spend a lot of time playing outside and chatting. I listen to Child 1 tell me about his day while I push Child 2 on the swing. He laughs a lot. Child 1 asks me why I wasn’t here last Monday. He’s noticing more when I’m not around!

I’m upstairs finishing my notes and preparing to leave. Child 9 calls me ‘auntie’ to ask me to play again. I tell him I must leave but we can play together next week. His sister takes him downstairs. The mother thanks me for playing with him.

Child 2 asks me to do something together. We painted our hands and then Child 3 joined us. I feel like the bond is there now.

Moreover, I also shared parts of my personal life with the children. I talked to them about my daughter, my dog, which also contributed to building connections with them, as the following excerpt highlights:

Child 3 arrives at the shelter. We have a snack together. I show her pictures of my dog. She wants to sit with me for the snack, wants to see the pictures of [dog], and asks me to listen to the song ‘Gummy Bear.’ I feel that these are signs that I’m starting to build a trusting connection with her.

Although these moments show signs of connection, reducing power imbalances was not immediate and should not be understood as an outcome, but rather as a process anchored in relationships with children and young people. Moments of refusal or distance, such as the one illustrated in the following excerpt, reminded me that children have the power to accept or reject my presence: “When saying goodbye to the younger girls, they are playing in the room and ask me to leave… Could this be linked to me being an adult? They want to play together.”

Respecting these moments of disconnection and allowing children to initiate interactions on their own terms did not necessarily eliminate adult power but created space for that power to be negotiated. This not only allowed for a deeper understanding of children’s experiences in shelters, but also, more broadly, of how trust and participation is built between adults and children.

These experiences embody a fundamental feminist principle aimed at reducing power imbalances between the shelter workers or researchers and the women they support (Corbeil & Marchand, 2010; Côté, 2017). This principle has been mostly applied to women. But as I will demonstrate in the discussion, feminist principles can also be applied to children.

## Discussion: Investing in relationships with children and young people

This paper aimed to show that, in research with children, it is not enough to reflect on adult power over them; it is also essential to implement methodological choices that address power dynamics and foster children’s experiences.

In line with the NSC, prioritizing children’s experiences allows for a more accurate understanding of their realities and supports better practices and policies (Sirota, 2006). It also helps reduce power imbalances and combat “adultism”-the oppression of children by adults in legal, social, political, and economic spheres Bell et al. (2018); Bell et al. (2018) argues that young people are among the most controlled groups in society and that their agency is often granted by adults. It is therefore up to adults to adapt to children.

Initial analysis of the field journal showed that I had invested significant time in building relationships with children and young people. Following the inductive approach of my research, I turned to the feminist ethics of care (Brannelly & Barnes, 2022) to make sense of this experience. In this section, I will show that while certain principles are essential in research with children, they are equally crucial in interventions with children in shelters -and that both fields can mutually inform each other.

Feminist care researchers stress the importance of studying topics researchers care about. Brannelly & Barnes (2022) argue that this connection should be valued, as it enriches understanding and strengthens ethical engagement. Creating inclusive spaces where participants feel safe and recognized is central (Brannelly and Barnes, 2022).

In this context, child-initiated play, showing interest in children’s and young people's interests and engaging in meaningful conversations contributed to a supportive research environment. Recognizing emotions in research is also crucial, allowing for deeper analyses of lived experiences. Moving away from objectivity, Brannelly & Barnes (2022) encourage a stance where empathy and reflexivity are key.

Feminist of care framework resonated with my ethnographic experience, given the time spent on building relationships, the emotions documented, and my critical reflection on my role with the children and on motherhood in shelter life.

### Power dynamics, feminist intervention, and research with children in shelters

For decades, feminists have reflected on and theorized feminist intervention with women in domestic violence shelters (Côté, 2017). Based on the findings presented in the previous section, I have highlighted several connections between feminist intervention and research with children, which I aim to explore further here.

#### Adapting to children’s worlds through play

Research on children’s experiences in shelters emphasizes that play is a crucial part of their everyday lives in these spaces (Chanmugam, 2011; Mullender et al., 1998; Thibault, 2021; Øverlien, 2011). Once they feel safe from violence, shelters provide a safe space where children have the freedom to engage in play (Mullender et al., 1998; Øverlien, 2011).

Investing time in building relationships with children and young people is a meaningful way to connect with them, particularly through play (Côté et al., 2020). This is especially true when play is initiated by the children themselves, as it allows adults to engage with them on their own terms, in the present moment - a moment that creates opportunities for meaningful connection with children (Corsaro, 2015). I integrated into their world by joining in the activities they led - running obstacle courses, engaging in imaginative play, singing their favorite songs, dancing, using available toys, pushing them on swings, drawing, cooking, and participating in special outings. As one mother pointed out, this may not align with a “traditional” approach to research, but from the perspective of the NSC - where children are seen as a marginalized group shaped by adult power - research with children requires doing things differently. It means engaging in activities that make sense to them, and play is one of the most effective ways to enter their world and reduce power imbalances between adults and children.

#### Building egalitarian relationships through the “least adult” posture

In addition to play, the “least adult” posture (Mandell, 2003) was particularly useful for establishing a connection with the children and young people and, above all, for reducing power dynamics. Adopting a “least adult” posture (Mandell, 2003) involves welcoming children and young people as they are, avoiding assumptions about their abilities, being mindful of judgments, and engaging in interactions in an enjoyable context, often through play (Côté et al., 2020). This posture led me to genuinely engage with the children’s and young people's interests without judgment, such as in relation to their use of screens, the language they used or when I engaged in their games and play.

In addition to the various actions highlighted through the “least adult” posture, one of the core principles of feminist intervention in shelters is to foster egalitarian relationships with the women residing there (Corbeil & Marchand, 2010; Côté, 2017). One way to achieve this is for workers to share their own experiences and perspectives with the women. This helps break down barriers between workers and women, thus strengthening their alliance. I applied this principle with the mothers by sharing my experiences of motherhood. I also did the same with the children: I talked about my daughter, showed them photos of my dog, and so on. Sharing with children, like with the women, can also be a way to address power dynamics.

While play was central to building connections with children, adopting a “least adult” posture has limitations. For example, I paid attention to family’s routines and shared time, such as mealtimes, and allowed them space to be together. I also respected workers interventions with children and the informal time they spent together. Conducting research in a shelter also required me to regularly clarify my role with children, as they sometimes perceived me as a worker. These boundaries supported an ethical way of conducting research in shelters, attentive to children, workers and mothers.

## Implications for practice

Drawing on feminist perspective and the NSC, this paper highlights the importance for practitioners to critically reflect on their power as adults and how it impacts children’s and young people’s everyday lives in shelters. Ethnographic attention to everyday interactions and routines makes visible how children experience shelter spaces, relationships, and everyday activities such as playing. Taking the time to invest in the relationships with children and young people through daily routines, being present in everyday moments and involving children and young people in the organization of daily life can support children’s participation and empowerment in the shelter and beyond.

## Conclusion

In conclusion, this paper highlights the importance of investing in relationships through a feminist ethics of care in research with children and young people. Beyond reflecting on adult power, it calls for methodologies that genuinely acknowledge and value children lived experiences. Ethnography offers a powerful tool to reduce power imbalances, fostering meaningful connections between researchers and children. These efforts are deeply tied to feminist intervention, aiming to transform unequal relationships and drive social change. Both research and intervention must challenge adultism by prioritizing relationships with children, ensuring their experiences are not only understood but fully valued.

## Notes

1. The term “child” refers to individuals aged 0 to 17 years.

2. The first-person perspective is used in this paper because the research was conducted by the first author, while the second author is the dissertation supervisor and contributed to the conceptual development and revision of the paper.

3. The practicum replaces the comprehensive exam and requires at least 450 hours in a practical setting, combined with academic activities. Its goal is to problematize and theorize an applied research project related to the field, by defining research questions. The objective is to train researchers who master the theories, practices, and policies of social work, while understanding their interactions and impacts in practical settings (Université d’Ottawa, 2017).
